# Pediatrics

**Published:** 1899-08

**Authors:** Maud J. Frye


### Progress in Medical Science.

### PEDIATRICS.

REPORTED BY MAUD J. FRYE, M. D.

### THE OXYURIS VERMICULARIS IN CHILDREN.

STILL (*British Med. Jour.*, April 15, 1899,) summarises his article, based on 200 necropsies on children under the age of twelve as follows:

1. The vermiform appendix is a common habitat of oxyuris vermicularis in childhood.

2. The generally accepted view that every single ovum of oxyuris vermicularis must be swallowed before it can be hatched is open to doubt, and there is a strong probability that the appendix vermiformis serves in some cases as a breeding place for threadworms.

3. The presence of threadworms in the appendix may cause a catarrhal condition therein, as shown post mortem, by a swollen appearance, due to thickening of its walls.

4. This swollen condition of the appendix, due to threadworms, is associated in some cases with pain in the right iliac fossa, whch may simulate ordinary appendicitis.

5. In the treatment of threadworms large injections must be used, and in view of the difficulty of dislodging the worms from the appendix, and their possible presence in the small intestine, the injections should be combined with the administration of drugs by the mouth.

---

### DIABETES MELLITUS IN CHILDREN.

TOWNSEND (*Boston Med. and Surg. Jour.*, May 11, 1899,) reports five cases of diabetes mellitus occurring in children under ten years of age and all terminating fatally, some with, some without treatment.

He notes the extreme rarity of the disease in childhood; the Massachusetts General Hospital in seventy-four years records 172 cases, in all of which only three were under ten years of age. Diabetes mellitus in infants is to be distinguished from the temporary glycosuria, which is sometimes seen in infants as the result of digestive disturbance and is not of serious import. He summarises the general picture of diabetes mellitus, as seen in children as follows:

The initial symptoms are apt to be incontinence of urine, due to polyuria; nervous irritability, great thirst. Strength, flesh and color may sometimes be retained until nearly the end. A gain of weight and height even may occur without any amelioration of the disease. The disease goes on to a fatal issue much more rapidly in children than in adults, the duration in my cases varying from two to fourteen months. Death is generally preceded by coma.

